# Evaluation of Antioxidant Potential of *Lavandula x intermedia* Emeric ex Loisel. ‘Budrovka’: A Comparative Study with *L. angustifolia* Mill.

**DOI:** 10.3390/molecules15095971

**Published:** 2010-08-30

**Authors:** Biljana Blažeković, Sanda Vladimir-Knežević, Adelheid Brantner, Maja Bival Štefan

**Affiliations:** 1 Department of Pharmacognosy, Faculty of Pharmacy and Biochemistry, University of Zagreb, Marulićev trg 20, 10000 Zagreb, Croatia; 2 Institute of Pharmaceutical Sciences, Department of Pharmacognosy, Karl-Franzens-University Graz, Universitaetsplatz 4, 8010 Graz, Austria

**Keywords:** *Lavandula x intermedia* Emeric ex Loisel. ‘Budrovka’, *Lavandula angustifolia* Mill., antioxidant activity, polyphenols, rosmarinic acid

## Abstract

A Croatian indigenous cultivar of lavandin, *Lavandula x intermedia* ‘Budrovka’ (Lamiaceae) was studied for the phenolic acids, flavonoids, anthocyanins, procyanidins and total tannins, as well as total polyphenols content, in the flower, inflorescence stalk and leaf ethanolic extracts. Antioxidant potentials on these plant part extracts were assessed by the DPPH free radical scavenging activity, iron chelating activity, reducing power, lipid peroxidation inhibition properties and total antioxidant capacity assays. All results were compared with those of *Lavandula angustifolia*, the only member of the *Lavandula* genus officially used in modern phytotherapy. Based on the results of our parallel study, we may suggest that *Lavandula x intermedia* ‘Budrovka’ is as potent an antioxidant as *Lavandula angustifolia* and the antioxidant activity of the *Lavandula* extracts is mainly due to the presence of rosmarinic acid. A good correlation was found between the polyphenol contents and antioxidant activities of the extracts.

## 1. Introduction 

Nowadays, medicinal plants are attracting considerable scientific interest as potential sources of natural antioxidants. Used as a nutritional dietary supplement/therapeutic agents, antioxidants are able to counteract oxidative stress and its deleterious effects on human health. In particular, a growing body of evidence suggests that oxidative stress, as an imbalance between free radical generation and elimination, is involved in the causation and progression of many diseases, including neuro-degenerative disorders, cardiovascular diseases, diabetes and cancer, as well as aging [1,2,3]. Because free radicals are highly reactive, they can cause damage of important biomolecules, such as lipids, proteins, DNA and RNA, leading to misfunction of these molecules and cell death [4]. Antioxidants can prevent those free radical-mediated oxidative damages by acting at different levels in the pathophysiological chain. Besides the health aspect, antioxidants are also used in food, cosmetic, and pharmaceutical products as additives which protect them against oxidative deterioration, such as lipid rancidity and related organoleptic changes with a consequent decrease in quality and safety caused by the formation of secondary, potentially toxic, compounds. Due to the toxic properties of widely used synthetic antioxidants, new, effective and safe natural antioxidant*s* are needed [5].

Former studies of plant-derived antioxidants have highlighted polyphenolic compounds as the most potent ones [5,6,7]. Polyphenols are a large group of secondary plant metabolites with highly diversified structures, and they can be categorized into several classes, such as tannins, flavonoids and phenolic acids. Prevention of oxidative damage through free radical scavenging activity, inhibition of their formation due to metal chelating properties as well as through the effects on cell signalling pathways and on gene expression are considered to be the primary mechanisms of their antioxidant action [8,9].

*Lavandula x intermedia* ‘Budrovka’ (Lamiaceae) is a Croatian indigenous cultivar of lavandin. This aromatic plant has been widely cultivated for its flowers which are used for the production of essential oil. In spite of the important economic value of lavandin ‘Budrovka’ its phytochemical constituents and biomedical potential have not yet been the subjects of any detailed study, although here are some old now dated reports on its essential oil composition [10,11]. Additionally, the antioxidant activity and phenolic content of lavandin was studied only for the waste remaining after distillation of the essential oil from an unspecified cultivar [12]. Therefore, the main objective of our work was to evaluate the potential of different plant parts of lavandin ‘Budrovka’, the flowers, inflorescence stalks and leaves, respectively, as a source of antioxidative agents, and to explore its relationship with the polyphenols present in extracts. All studies were conducted in comparison with *Lavandula angustifolia,* the only member of the genus *Lavandula* which is officially recognised as a medicinal plant and used in modern phytotherapy. On the other hand, our literature survey revealed that there is still a lack of detailed data on the polyphenolic contents as well as the antioxidant activities of *L. angustifolia* [13,14,15,16,17,18]. Therefore, in accordance with the conclusions of Sariri *et al*. [19], more extensive studies of *L. angustifolia* and related taxa are still warranted.

## 2. Results and Discussion 

### 2.1. Contents of polyphenols and HPTLC of phenolic acids

It is well known that polyphenols are important plants constituents, and they have attracted a great deal of scientific interest due to their health promoting effects as antioxidants. Considering their wide structural diversity, in our study the contents of different polyphenolic subclasses were determined in the investigated ethanolic extracts of *Lavandula x intermedia* ‘Budrovka’ and *L. angustifolia*, and the results of analyses are summarised in Table 1. In general, the contents of most of the polyphenolic constituents varied greatly among individual plant parts, flowers, inflorescence stalks and leaves, respectively, as well as between the two *Lavandula* taxa studied. The leaf extracts had higher total amounts of phenolic acids, flavonoids, procyanidins, total tannins as well as total polyphenols than the corresponding flower and inflorescence stalk extracts. Noticeably, presence of anthocyanins (0.02–0.03%) was only determined in the flower extracts. 

The most abundant compounds found in the *Lavandula* extracts were phenolic acids, ranging between 1.62% and 3.80% for *L*. *x intermedia* ‘Budrovka’ and from 2.41% to 5.32% for *L. angustifolia* extracts, respectively. The leaf extracts were the richest sources of phenolic acids, followed by flowers and inflorescence stalk extracts. Flavonoids contents in *Lavandula* extracts ranged from 0.09% for flower extract to 0.26% for leaf extract. There were no significant differences in flavonoids contents among the extracts of *L. x intermedia* ‘Budrovka’ and *L. angustifolia* (P > 0.05). The contents of total tannins determined in *L. x intermedia* ‘Budrovka’ extracts differ significantly (P < 0.001) decreasing in order leaf (2.21%), flower (2.02%) and inflorescence stalk (1.01%). Extracts obtained from *L. angustifolia* contained significantly (P < 0.05) higher amount of tannins, 3.18%, 2.77% and 1.38%, respectively, following the same order of plant parts. The percentage of procyanidin group of tannins varied between 0.86% and 1.44% for all *Lavandula* extracts. Total polyphenol content of *L. x intermedia* ‘Budrovka’ ethanolic extracts differed greatly and was highest in leaf (7.05%), followed by flower (6.65%) and inflorescence stalk (3.09%) extracts (P < 0.05). However, similar extracts obtained from *L. angustifolia* contained significantly (P < 0.001) greater quantity of total polyphenols, 9.20%, 8.46% and 4.54%, namely. These amounts were considerably higher than the values found previously in other *L. angustifolia* extracts (0.5-2.6%) [13,16,17]. 

**Figure 1:**
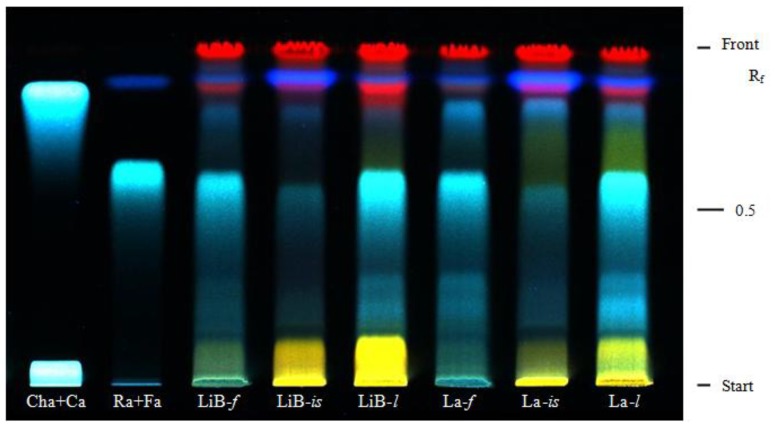
HPTLC chromatogram of phenolic acids in *Lavandula x intermedia* ‘Budrovka’ and *L. angustifolia* extracts.

The presence of phenolic acids in tested *Lavandula* extracts was detected by high performance thin layer chromatography (HPTLC). For all extracts screened blue zones with R_f_ values of 0.62, 0.86 and 0.89 were detected (Figure 1) and identified, in comparison with related standards, as rosmarinic, caffeic and ferulic acids, respectively. The fluorescence intensity of the zones varied greatly among individual plant organs. The zones corresponding to rosmarinic acid were very intense for leaves and flowers of both *Lavandula* taxa, but very weak for inflorescence stalks which were characterised by more intensive zones corresponding to ferulic acid. These findings were consistent with the results obtained by quantitative analyses, indicating the greatest contribution of rosmarinic acid to the total phenolic acid contents. 

### 2.2. Antioxidant activity of Lavandula x intermedia *‘Budrovka’ and* L. angustifolia *extracts*

Natural antioxidants can inhibit the initiation or propagation of undesirable oxidation chain reactions by exerting their action through different mechanisms, including free radical-scavenging, reducing activity and complexing of pro-oxidant metal ions [20]. Therefore, in the present study five different *in vitro* assays were employed in order to determine and compare the antioxidant properties of *Lavandula x intermedia* ‘Budrovka’ and *L. angustifolia*, as well as to elucidate their mode of action. 

**Figure 2:**
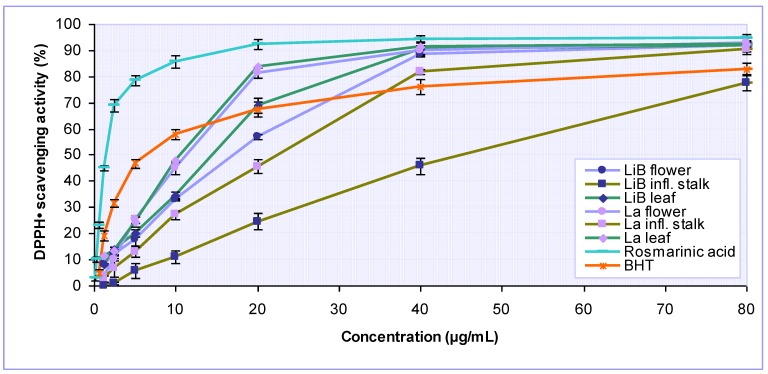
DPPH free radical scavenging activity of various *Lavandula x intermedia* ‘Budrovka’ (LiB) and *L. angustifolia* (La) extracts as well as rosmarinic acid at different concentrations. BHT was used as reference.

The free-radical scavenging activities of investigated extracts were evaluated by the commonly used DPPH method. DPPH is as a stable free radical that can accept an electron or hydrogen radical from the antioxidant agents to become a stable diamagnetic molecule. As can be seen in Figure 2, all investigated *Lavandula* extracts exhibited a potent antiradical activity in a concentration dependent manner. The effectiveness of *L. x intermedia* ‘Budrovka’ and *L. angustifolia* extracts decreased in the following order: leaf > flower > inflorescence stalk. At higher concentrations (40–160 µg/mL) most of the tested extracts were more effective in DPPH radical scavenging than the widely used synthetic antioxidant BHT, while at lower concentrations (≤ 10 µg/mL) BHT was considerably more potent. Calculated concentrations of tested samples needed to scavenge 50% of DPPH^·^ (IC_50_) are given in the Table 2. IC_50_ values for *L. x intermedia* ‘Budrovka’ extracts ranged from 15.06 µg/mL to 45.25 µg/mL, while those obtained for extracts of *L. angustifolia* were significantly (P < 0.001) lower (IC_50_ 10.62–33.95 µg/mL), reflecting their better free radical scavenging activity. 

In contrast to the inconsistent results of previously reported studies on *L. angustifolia*, our study highlighted very strong and statistically not different antiradical activities of leaf and flower ethanolic extracts (IC_50_ 11.37 and 10.62 µg/mL). Namely, the previous single-concentration studies by Ferreira *et al*. [15] showed that ethanolic extract from flowering aerial parts inhibited 23% of DPPH free radicals at 100 µg/mL, Tsai *et al.* [17] reported very low DPPH antiradical activity (6.15%) of flower methanolic extract at 1000 µg/mL, while Sariri *et al.* [19] determined an IC_50_ value of 29.2 µg/mL for aqueous leaf and flower extract. Rosmarinic acid was found to be an important constituent of the *Lavandula* extracts. Although some previous studies indicated that rosmarinic acid is a potent antioxidant, reported IC_50_ values varied widely, from 2.9 µg/mL to 41.3 µg/mL [21,22,23]. Our results demonstrated that rosmarinic acid is a very strong DPPH radical scavenger with IC_50_ value of 1.51 µg/mL, being even four times more effective than positive control BHT (IC_50_ 6.45 µg/mL). Based on the results of our parallel study, we may suggest that the noticeable antiradical efficiency of the *Lavandula* extracts is greatly affected by the presence of this phenolic acid. 

**Figure 3:**
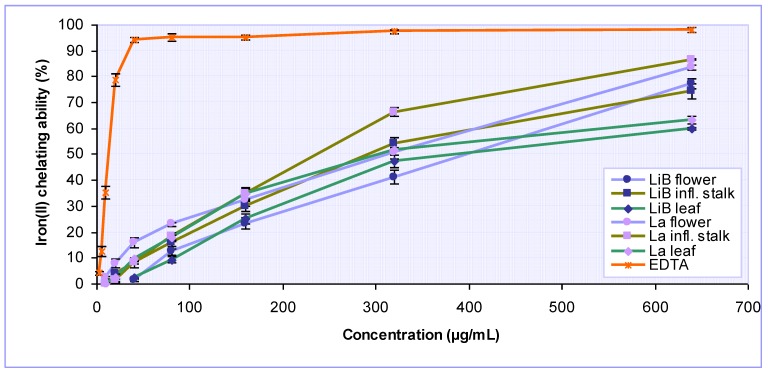
Iron(II) ions chelating activity of various *Lavandula x intermedia* ‘Budrovka’ (LiB) and *L. angustifolia* (La) extracts at different concentrations. EDTA was used as reference.

Transition metal ions, such as those of iron(II) and copper, have the potential to mediate deleterious oxidations of biomolecules (lipids, proteins, DNA and others). The binding of transition metal ions by chelating agents can stabilize prooxidative activity of those ions [24]. Because of the importance of metal chelation as one of the antioxidant activity mechanisms, the ability of the *Lavandula* extracts to compete with ferrozine for iron ions in free solution was studied, and the results obtained are graphically presented in Figure 3, while the corresponding IC_50_ values are given in Table 2. All the *Lavandula* extracts were shown to possess significant and concentration-dependent iron-chelating activity. Among the different plant parts, inflorescence stalk extracts of both *Lavandula* plants were found to be the significantly (P < 0.001) more active then related flower and leaf extracts, respectively. The effectiveness of plant extracts as iron ions chelators was in the following descending order: *L. angustifolia* inflorescence stalk > *L. x intermedia* ‘Budrovka’ *inflorescence* stalk > *L. angustifolia* leaf > *L. angustifolia* flower > *L. x intermedia* ‘Budrovka’ leaf > *L. x intermedia* ‘Budrovka’ flower, with IC_50_ values ranged between 236.92 and 397.71 µg/mL. However, the chelating ability of all tested extracts was much lower compared to reference EDTA, which is one of the most powerful metal chelators known (IC_50_ 13.37 µg/mL). Contrary to the extracts, neither rosmarinic acid nor BHT showed any iron-chelating activity at the concentrations studied (shown on Table 2). 

**Figure 4:**
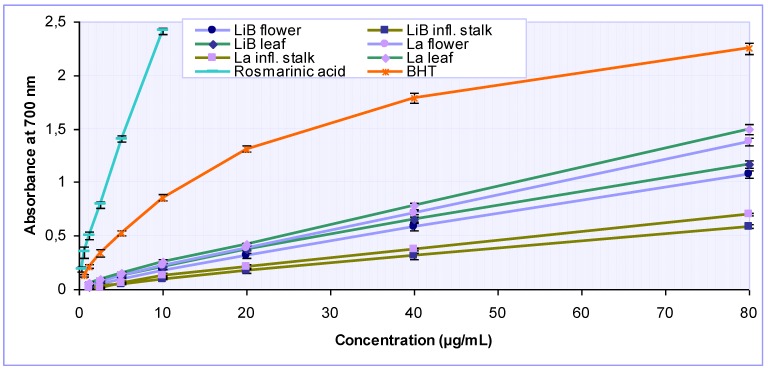
Reducing power of various *Lavandula x intermedia* ‘Budrovka’ (LiB) and *L. angustifolia* (La) extracts as well as rosmarinic acid at different concentrations. BHT was used as reference.

Various studies have indicated that the reductive capabilities of natural antioxidants, in terms of iron(III)-iron(II) transformation, are closely related with their activity [25,26]. The reducing property is generally associated with the presence of reductones which exert antioxidant action by breaking the free radical chains via hydrogen atom donation. Besides, reductones can also prevent peroxide formation by reacting with certain precursors. Figure 4 depicts the reducing power for different concentrations of *Lavandula* extracts, their constituent rosmarinic acid and BHT as a positive control. Our results demonstrated that all tested samples possessed the ability to reduce iron(III) ions. The reducing power of the *Lavandula* extracts increased with a concentration in a strongly linear manner (R^2^ = 0.9938-0.9994). For the *L. x intermedia* ‘Budrovka’, the leaf extract showed the strongest reducing ability with IC_50_ 28.73 µg/mL, followed by the flower extract (IC_50_ 33.78 µg/mL) and inflorescence stalk extract (IC_50_ 66.92 µg/mL), but all extracts were significantly (P < 0.001) less active then related *L. angustifolia* extracts (Table 2). The reducing power of rosmarinic acid (IC_50_ 1.26 µg/mL) was considerably more pronounced relative to that of BHT (IC_50_ 4.64 µg/mL), suggesting its strong influence on the reductive properties of *Lavandula* extracts. 

**Figure 5:**
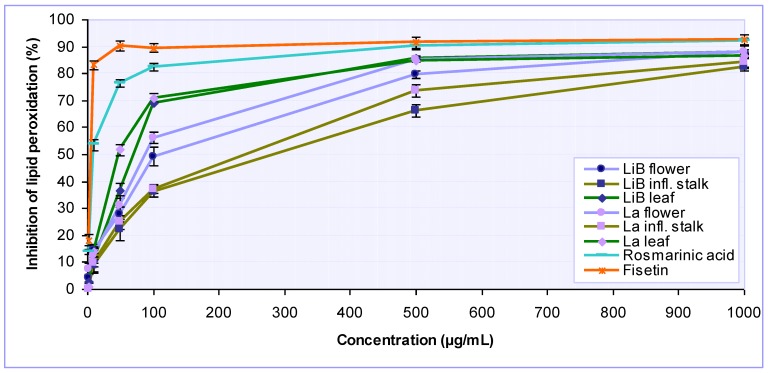
Inhibition of lipid peroxidation by various *Lavandula x intermedia* ‘Budrovka’ (LiB) and *L. angustifolia* (La) extracts as well as rosmarinic acid at different concentrations. Fisetin was used as reference.

Among all biological macromolecules, unsaturated membrane lipids are particularly prone to oxidative damage. Therefore, the lipid peroxidation, as a well-established mechanism of cellular injury, can be used as an indicator of oxidative stress in cells and tissues [27]. In the present study we measured the potential of investigated *Lavandula* extracts and its constituent rosmarinic acid to inhibit lipid peroxidation induced by the Fe(II)–H_2_O_2_ system in bovine brain liposomes [28]. The extent of lipid peroxidation was evaluated in terms of production of thiobarbituric acid-reactive substances (TBARS) and the results obtained are presented in Figure 5 and Table 2. All tested *Lavandula* extracts inhibited lipid peroxidation in a concentration-dependent manner. At a concentration of 100 μg/mL, the flower, inflorescence stalk and leaf extracts of *L. x intermedia* ‘Budrovka’ effected 49.30%, 36.14% and 69.21% inhibition of lipid peroxidation activity, while the extracts from *L. angustifolia* effected 56.17%, 37.20% and 70.75%, respectively. Generally, *Lavandula* extracts were less potent then fisetin and rosmarinic acid. However, at the highest tested concentration 1000 µg/mL plant extracts almost reached the activities of pure compounds. Leaf extracts of *L. angustifolia* and *L. x intermedia* ‘Budrovka’ displayed stronger activity then extracts from other plant parts and proved to be able to prevent lipid peroxidation in significantly (P < 0.001) lower concentrations (IC_50_ 54.57 and 74.56 µg/mL, respectively). 

**Figure 6:**
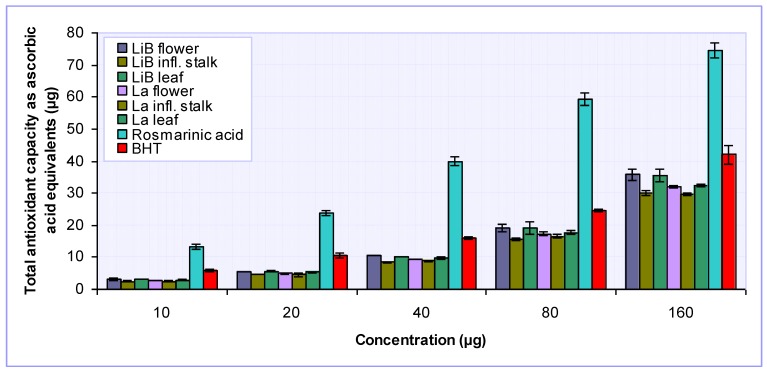
Total antioxidant capacity of various *L. x intermedia* ‘Budrovka’ (LiB) and *L. angustifolia* (La) extracts and rosmarinic acid. BHT was used for comparison.

Total antioxidant capacities of investigated *Lavandula* extracts, rosmarinic acid and reference antioxidant (BHT) were evaluated by phosphomolybdenum assay, based on the reduction of Mo(VI) to Mo(V) by the antioxidant compounds and the subsequent formation of a green phosphate/Mo(V) complex at acidic pH [29]. The results obtained, expressed as ascorbic acid equivalents (AAE), are presented in Figure 6 and Table 2. 

Among *Lavandula* extracts, the highest total antioxidant capacities were determined for the flower and leaf extracts of *L. x intermedia* ‘Budrovka’, with approximately equal values of 294.00 and 290.75 mg AAE expressed per gram of dry extract. The extracts from the same plant parts of *L. angustifolia* displayed significantly (P < 0.05) lower antioxidant properties (261.50 and 274.18 mg AAE/g dry extract, respectively). Interestingly, these findings were in contrast to the above-mentioned results obtained by other assays. The lowest antioxidant capacities were found for the inflorescence stalk extracts (214.96 and 218.08 mg AAE/g, respectively) and they were not significantly (P > 0.05) different among two *Lavandula* taxa studied. Although the total antioxidant capacity of BHT was found to be much lower (P < 0.001) when compared to rosmarinic acid, none of the tested *Lavandula* extracts was as effective as the reference antioxidant. This assay also confirmed that rosmarinic acid is an important contributor to the overall antioxidant capacity in *Lavandula* extracts, as previously suggested by other antioxidant assays used in our study. Additionally, the presented results imply that the extracts obtained from flowers and leaves of *L. x intermedia* ‘Budrovka’ have a strong ability to act as antioxidant as compared to *L. angustifolia* extracts.

### 2.3. Correlations between polyphenols and antioxidant activity

In order to elucidate the influence of polyphenolic constituents on the antioxidant activity of Lavandula extracts, a correlation study between the results obtained in different antioxidant assays and determined contents of different classes of polyphenols was performed. Presented r values (Table 3), ranging from 0.9017 to 0.9936, revealed very strong and significant positive correlation between the total phenolic acid, procyanidin, total tannin as well as total polyphenol contents with most of the antioxidant assays, such as antiradical activity (P < 0.01), reducing power (P < 0.01) and inhibition of lipid peroxidation (P < 0.05). Our findings were consistent with the previous reports on significant contribution of the polyphenols to the antioxidant activity of medicinal plants [30,31]. However, total antioxidant capacity showed only a moderate positive correlation with the four above-mentioned polyphenolic groups (*r* 0.4643-0.5625), indicating that some other phytochemicals also contribute to the total antioxidant capacity of *Lavandula* extracts. In our study very weak and statistically nonsignificant relationship was found between the flavonoid content and all determined antioxidant properties of *Lavandula* extracts. A similar result was also observed by Moein *et al.* [32]. Likewise, iron chelating abilities of the tested extracts can’t be related to the presence of a polyphenols.

## 3. Experimental 

### 3.1. Plant material and extraction procedure

Aerial parts of *Lavandula x intermedia* Emeric ex Loisel. ‘Budrovka’ and *Lavandula angustifolia* Mill. were collected at full flowering stage in July 2006 from cultivated plants at the farm in the village Dragovanščak, near to the city Jastrebarsko (Central Croatia, 45°70′ N, 15°55′ E). The heights of five-year-old plants of *L. x intermedia* ‘Budrovka’ and *Lavandula angustifolia* were 105 cm and 60 cm, respectively. Plant material was air-dried and leaves, flowers and inflorescence stalks were separated. The plant samples were identified at the Department of Pharmacognosy, Faculty of Pharmacy and Biochemistry, University of Zagreb, Croatia, where the voucher specimens (No. FBF-FGN/BB 101 and 102) have been deposited. 

Pulverized plant material (30 g) was extracted twice with a total of 500 mL of 80% ethanol using an ultrasonic bath (Sonorex digital 10P, Bandelin Electronic, Germany) for 30 minutes. After filtration through Whatman No. 1 filter paper (Whatman International Ltd., UK) in a Buchner funnel, the obtained extract was concentrated to dryness under vacuum at 50 ºC using a rotary evaporator. The yields of extracts (w/w) from flower, inflorescence stalk and leaf of *L. x intermedia* ‘Budrovka’ were 23.9%, 14.6% and 20.8%, while for *L. angustifolia* they amounted 14.8%, 10.9% and 14.2%, respectively.

### 3.2. Chemicals

Aluminium chloride, ethylenediaminetetraacetic acid (EDTA), hexamethylenetetramine, sodium carbonate, sodium citrate, sodium hydroxide, sodium nitrite and sodium phosphate were purchased from Kemika (Zagreb, Croatia). Ammonium molybdate, bovine brain extract (Folch type VII), caffeic acid (≥98%), chlorogenic acid (≥95%), ferulic acid (99%), 3-(2-Pyridyl)-5,6-diphenyl-1,2,4-triazine-4′,4′′-disulfonic acid sodium salt (ferrozine), 2,2-diphenyl-1-picryl-hydrazyl (DPPH), potassium ferricyanide, pyrogallol (99%), rosmarinic acid (96%), sodium acetate and sodium molybdate were obtained from Sigma-Aldrich (St. Louis, MO, USA). Butylhydroxytoluene (BHT; ≥99%), 2-aminoethyl diphenylborinate (natural product reagent) and iron(II) chloride were obtained from Fluka (Buchs, Switzerland). Ascorbic acid (99%) and trichloroacetic acid were purchased from Acros Organics (Geel, Belgium), Folin–Ciocalteu’s phenol reagent and 2-thiobarbituric acid were obtained from Merck (Darmstadt, Germany). Iron(III) chloride and fisetin (≥99%) were obtained from Riedel-de Haën (Seelze, Germany) and Carl Roth (Karlsruhe, Germany), respectively. Other reagents and solvents used were of analytical grade.

### 3.3. Phytochemical analyses of polyphenols

Determination of phenolic acids in plant extracts was performed according to procedure described in European Pharmacopoeia [33]. Briefly, the extract (0.2 g) was boiled with 50% ethanol (80 mL) for 30 min in a water bath under a reflux condenser. The cooled extract was filtered, the filter rinsed with ethanol, and then combined filtrate and rinsing were diluted to 100 mL with 50% ethanol. An aliquot (1 mL) of the extract was mixed with 0.5 M hydrochloric acid (2 mL), Arnow reagent (10% aqueous solution of sodium nitrite and sodium molybdate, 2 mL), and 8.5% sodium hydroxide (2 mL) and diluted to 10 mL with water. The absorbance of the test solution was measured immediately at 505 nm against sample blank. The content of phenolic acids, expressed as rosmarinic acid, was calculated according to the following expression: *(%) = A*×*2.5*/*m*, where *A* is the absorbance of the test solution at 505 nm and *m* the mass of the sample, in grams.

Determination of flavonoid content in the investigated extracts was performed according to the method of Christ and Müller [34]. One mL of 0.5% hexamethylenetetramine solution, acetone (20 mL) and 25% hydrochloric acid (2 mL) were added to the extract sample (0.5 g) and set to boil with reflux for 30 min. After filtration, the residue was re-extracted twice with acetone (20 mL) for 10 min and the volume of filtrates was completed to 100 mL with acetone. An aliquot (20 mL) of the acetone extract was put into a separation funnel, along with water (20 mL) and ethyl acetate (15 mL). The extraction with ethyl acetate was carried out three times and the combined ethyl acetate layers were then washed twice, using 20 mL of water each time, and were subsequently diluted to 50 mL with ethyl acetate. The total flavonoid content was determined in 10 mL of this solution, using 2% aluminium chloride solution (in 5% methanolic solution of acetic acid, 2 mL), 0.5% sodium citrate (5 mL) and the mixture was made up to 25 mL with 5% methanolic solution. After 45 min, the absorbance was read at 425 nm, and the percentage content of flavonoids, expressed as quercetin, was calculated from the equation: *(%) = A×0.772*/*b*, where *A* is the absorbance of the test solution at 425 nm and *b* the mass of the sample, in grams.

Determination of anthocyanins in the extracts was conducted according to European Pharmacopoeia method [33]. In short, methanol (95 mL) was added to the extract and after mechanical stirring for 30 min, the mixture was filtrated and diluted to 100 mL. 50-fold dilution of this solution was prepared using a 0.1% hydrochloric acid in methanol, and the absorbance of obtained solution was measured at 528 nm. The percentage content of antocyanins, expressed as cyanidin-3-glucoside chloride, was calculated from the expression: *(%) = A×5000/718×m*, where *A* is the absorbance at 528 nm, *718* specific absorbance of cyanidin-3-glucoside chloride at 528 nm, and *m* the mass of the examined extract, in grams. 

Determination of procyanidin content was done using European Pharmacopoeia method [33]. Briefly, 70% ethanol (30 mL) was added to the extract (2.5 g), heated under reflux condenser for 30 min and then filtered. The obtained filtrate was then mixed with hydrochloric acid (15 mL) and water (10 mL), and then heated for 80 min. After cooling and filtrating, the filtrate was made up to 250 mL with 70% ethanol. Fifty mL of this solution was transferred to separating funnel and shaken with three portions of butanol (each of 15 mL). The combined organic layers were diluted to 100 mL with butanol, and the absorbance of the solution was measured at 545 nm. The percentage content of procyanidins was calculated as cyanidin chloride from the expression: *(%) = A×6.7/m*, where *A* is the absorbance at 545 nm, and *m* the mass of the examined extract, in grams. 

Determination of total tannin as well as total polyphenol contents was performed following the method described in European Pharmacopoeia [33]. Briefly, the extract (0.5 g) was boiled for 30 min in a water bath with water (150 mL), then the filtrate was made up to 250 mL with water and the obtained solution served as stock solution. An aliquot of stock solution was mixed with Folin–Ciocalteu’s phenol reagent and sodium carbonate solution. After 30 min, the absorbance was read at 760 nm (A_1_), and the quantification of total phenols was done with respect to the standard calibration curve of pyrogallol (6.25–50.00 mg). For the determination of tannins content, stock solution was vigorously shaken with hide powder for 60 min. Since the hide powder adsorbed tannins, phenols unadsorbed on hide powder were measured in filtrate, after addition of Folin–Ciocalteu’s phenol reagent in a sodium carbonate medium (A_2_). The percentage content of tannins, expressed as pyrogallol, was calculated from the following equation: *(%) = 3.125×(A_1_-A_2_)/(A_3_×m),* where *A_3_* is the absorbance of the test solution containing 0.05 g of pyrogallol, and *m* the mass of the extract (g).

High performance thin-layer chromatographic (HPTLC) analysis of phenolic acids was performed on precoated silica gel 60 F_254_ HPTLC plate (Merck, Germany). Ethanolic extracts and phenolic acid standards solutions (30 μL) were applied on the plate using Automatic TLC sampler 4 (Camag, Switzerland) and developed using a mixture of ethyl acetate:acetic acid (95:5 V/V) as mobile phase [35]. After the run the plate was sprayed with natural products-polyethylene glycol reagent (1% methanolic solution of 2-aminoethyl diphenylborinate and 5% ethanolic solution of PEG 4000) and detection was carried out under UV (365 nm) by use of a Reprostar 3 System (Camag, Switzerland).

### 3.4. Evaluation of antioxidant activity

#### 3.4.1. Determination of DPPH free radical-scavenging activity

Free radical scavenging activities of the samples were measured using the stable DPPH radical as described by Blois [36]. Briefly, ethanolic DPPH·solution (1 mL, 0.1 mM) was added to sample solutions (3 mL) of different concentrations (0.63–160 µg/mL). The mixtures were shaken vigorously and incubated for 30 min in the dark at room temperature. Absorbance was measured at 519 nm. Butylated hydroxytoluene (BHT) was used as a positive control. DPPH^·^ scavenging effect was calculated as follows: *(%) = [(A_Control_–A_Sample_)/A_Control_]×100,* where *A_Control_* is the absorbance of the control reaction and *A_Sample_* is the absorbance in the presence of sample, corrected for the absorbance of sample itself.

#### 3.4.2. Determination of iron chelating activity

The chelation of iron(II) ions by the tested samples was estimated by a reported method [25]. Different concentrations of the sample (20–640 μg/mL final concentration) were added to a solution of 2 mM iron(II) chloride (0.05 mL). The reaction was initiated by the addition of 5 mM ferrozine (0.2 mL) and the mixture was finally quantified to 4 mL with ethanol, shaken vigorously and left standing at room temperature for 10 min. Absorbance of the solution was measured spectrophotometrically at 562 nm. EDTA was used as a reference chelator. The percentage inhibition of ferrozine–Fe(II) complex formation was calculated using the formula: *(%) = [A_0_-(A_1_-A_2_)]/A_0_ × 100*, where *A*_0_ is the absorbance of the control, containing iron(II) chloride and ferrozine, *A*_1_ is the absorbance in the presence of the test sample and *A*_2_ is the absorbance of the sample under identical conditions as *A*_1_ with water instead of iron(II) chloride solution.

#### 3.4.3. Determination of reducing power

The ability of the tested samples to reduce iron(III) ions was assessed as previously described [25]. An aliquot (1.0 mL) of sample at various concentrations (1.25–80 μg/mL) was mixed with 0.2 M phosphate buffer (pH 6.6, 2.5 mL) and 1% potassium ferricyanide (2.5 mL). The mixture was incubated at 50 ºC for 20 min. Subsequently, 10% trichloroacetic acid (2.5 mL) was added and the mixture was centrifuged for 10 min. The supernatant (2.5 mL) was mixed with distilled water (2.5 mL) and 1% iron(III) chloride (0.5 mL), and the absorbance of the mixture was read at 700 nm. Higher absorbance of the reaction mixture indicated greater reducing power. The sample concentration providing absorbance of 0.5 (IC_50_) was calculated from the graph of absorbance at 700 nm against sample concentration. BHT was used for comparison.

#### 3.4.4. Determination of inhibition of lipid peroxidation 

The ability of the extracts to inhibit lipid peroxidation was evaluated using the phospholipid liposomes prepared from bovine brain extract, according to previously described method [28] with minor modifications. Tested samples were dissolved in DMSO to give final concentrations in the reaction mixtures in the range of 1–1000 μg/mL. Bovine brain extract (Folch type VII) was mixed with 10 mM phosphate-buffered saline (pH 7.4) and sonicated in an ice bath until an opalescent suspension was obtained containing 5 mg/mL phospholipid liposomes. The reaction mixture contained the liposomal suspension (0.5 mL), tested sample (0.01 mL), iron(III) chloride (1 mM, 0.1 mL) and phosphate buffer (0.3 mL). The peroxidation was initiated by adding 1 mM ascorbic acid (0.1 mL). All reagents were prepared freshly. After incubation for 60 min at 37 ºC the extend of peroxidation was determined using the TBA test: 2-thiobarbituric acid (1% in 0.05 M sodium hydroxide, 1 mL), 2.8% trichloroacetic acid (1 mL) and 2% BHT (0.1 mL) were added and the tubes were heated in a water bath at 80 ºC for 20 min. After cooling and centrifugation, the absorbance was read at 532 nm. Control tubes included liposomes subjected to the TBA test without addition of Fe-ascorbate (blanks) as well as tubes lacking test sample (negative control reaction). Inhibition of lipid peroxidation in percent was calculated using the following equation: *(%) = [(A_Control_–A_Sample_)/A_Control_]×100*, where *A_Control_* was the absorbance of the negative control reaction and *A_Sample_* was the absorbance in the presence of the tested sample. Fisetin was used as a positive control. 

#### 3.4.5. Determination of total antioxidant capacity

Total antioxidant capacities of the tested samples were determined by the phosphomolybdenum method, according to the procedure of [29]. Briefly, an aliquot (0.4 mL) of sample solution of different concentration (10–160 μg/mL) was combined with reagent solution (0.6 M sulfuric acid, 28 mM sodium phosphate and 4 mM ammonium molybdate, 4 mL). The reaction mixture was incubated in a water bath at 95 ºC for 90 min. After cooling to room temperature, the absorbance was measured at 695 nm. The antioxidant capacity of the sample was expressed as equivalents of ascorbic acid (AAE), utilizing a calibration curve of ascorbic acid in the concentration range 1.25–160 μg/mL.

### 3.5. Statistical analysis

All experiments were carried out in triplicate, and the results are expressed as mean ± standard deviation (s.d.). Differences were estimated by Student’s t-test and the values P < 0.05 were considered statistically significant. The concentrations of samples that provide 50% inhibition (IC_50_) were obtained by interpolation from linear regression analysis. Correlation analyses between the various antioxidant assays and phenolic contents were performed calculating the Pearson’s correlation coefficient (*r*). All statistical analyses were carried out using Microsoft Excel 2000 software. 

## 4. Conclusions 

Our comprehensive study reports for the first time the detailed polyphenolic contents of the individual plant parts of two economically important *Lavandula* taxa, *L. x intermedia* ‘Budrovka’ and *L. angustifolia*, as well as the antioxidant properties of their extracts. Comparing the effectiveness of flower, leaf and inflorescence stalk extracts, we found that the leaf extracts of both *Lavandula* taxa were mainly the most active, closely followed by flower extracts, while the inflorescence stalk extracts exerted lower antioxidant activity. Most of the antioxidant assays used, except determination of total antioxidant capacity, revealed that *L. x intermedia* ‘Budrovka’ extracts were slightly less potent in relation to the extracts from *L. angustifolia*, what can be attributed to their lower polyphenolic contents. Namely, obtained correlation coefficients exhibited a strong positive association among different modes of antioxidant action (antiradical activity, reducing power and lipid peroxidation inhibition) and contents of phenolic acids, procyanidins, total tannins and total polyphenols in all tested *Lavandula* extracts, indicating that phenolic compounds could be major contributors to their antioxidant properties. Rosmarinic acid, as the most abundant polyphenolic constituents of tested *Lavandula* extracts, is very likely to largely contribute to observed antioxidant effects. In conclusion, our results indicated that both closely related *Lavandula* taxa could be valuable sources of natural antioxidants.

## Figures and Tables

Cha = chlorogenic acid (R_f_ 0.06); Ca = caffeic acid (R_f_ 0.86); Ra = rosmarinic acid (R_f_ 0.62); Fa = ferulic acid (R_f_ 0.89); LiB*-f* = flower, LiB*-is* = inflorescence stalk, and LiB*-l* = leaf extract from *L. x intermedia* ‘Budrovka’, respectively; La*-f* = flower, La*-is* = inflorescence stalk, and La*-l* = leaf extract from *L. angustifolia,* respectively.

**Table 1:** Contents of phenolic acids, flavonoids, anthocyanins, procyanidins, total tannins and total polyphenols in the various *Lavandula x intermedia ‘Budrovka’ (LiB)* and *L. angustifolia (La)* extracts.

Extracts		Contents (%)					
		Phenolic acids	Flavonoids	Anthocyanins	Procyanidins	Total tannins	Total polyphenols
*LiB*	flower	3.42 ± 0.09	0.10 ± 0.01	0.02 ± 0.00	1.13 ± 0.07	2.02 ± 0.01	6.65 ± 0.14
	stalk	1.62 ± 0.12	0.22 ± 0.01	-	0.86 ± 0.02	1.01 ± 0.04	3.09 ± 0.11
	leaf	3.80 ± 0.04	0.26 ± 0.01	-	1.30 ± 0.05	2.21 ± 0.03	7.05 ± 0.15
							
*La*	flower	5.00 ± 0.11	0.09 ± 0.01	0.03 ± 0.00	1.32 ± 0.08	2.77 ± 0.05	8.46 ± 0.05
	stalk	2.41 ± 0.06	0.19 ± 0.02	-	1.02 ± 0.03	1.38 ± 0.19	4.54 ± 0.22
	leaf	5.32 ± 0.14	0.25 ± 0.01	-	1.44 ± 0.02	3.18 ± 0.22	9.20 ± 0.17

Each value is the mean ± s.d. of triplicate measurements; - not determined.

**Table 2:** IC_50_ values of various *Lavandula x intermedia ‘Budrovka’* (*LiB*) and *L. angustifolia* (*La*) extracts, their constituent rosmarinic acid and references.

Extracts		IC_50_^*^ (µg/mL)				Total antioxidant capacity (mg AAE/g)
		DPPH^·^ scavenging activity	Iron chelating activity	Reducing power	Inhibition of lipid peroxidation	
*LiB*	flower	17.17 ± 0.33	397.71 ± 10.26	33.78 ± 2.34	116.54 ± 9.96	294.00 ± 13.17
	infl. stalk	45.25 ± 0.10	294.08 ± 12.86	66.92 ± 3.75	283.54 ± 5.02	240.75 ± 13.08
	leaf	15.06 ± 0.74	383.59 ± 15.55	28.73 ± 1.61	74.56 ± 4.71	290.75 ± 8.99
					
*La*	flower	11.37 ± 0.69	319.21 ± 21.96	25.17 ± 0.16	89.36 ± 5.00	261.50 ± 7.07
	infl. stalk	33.95 ± 1.42	236.92 ± 10.19	55.22 ± 1.10	240.48 ± 11.00	238.08 ± 8.38
	leaf	10.62 ± 0.02	302.79 ± 7.61	24.26 ± 0.76	54.57 ± 4.42	274.18 ± 5.07
					
Rosmarinic acid		1.51 ± 0.07	NA	1.26 ± 0.14	9.18 ± 0.01	1064.47 ± 53.52
BHT		6.45 ± 0.53	NA	4.64 ± 0.31	-	414.74 ± 36.81
EDTA		-	13.37 ± 0.91	-	-	
Fisetin		-	-	-	5.42 ± 0.60	

^*^ IC_50_ value = concentration at which the DPPH radicals were scavenged by 50%, iron(II) ions were chelated by 50%, absorbance was 0.5 for reducing power and lipid peroxidation was inhibited by 50%, respectively; Each value is expressed as mean ± s.d. (n = 3); NA = not active; - = not tested.

**Table 3:** The *r* values (Pearson’s correlation coefficient) between antioxidant activities and polyphenolic contents.

	DPPH·scavenging activity	Iron chelating activity	Reducing power	Inhibition of lipid peroxidation	Total antioxidant capacity
**Phenolic acids**	0.9936^**^	−0.3425^ns^	0.9804^**^	0.9017^*^	0.4643^ns^
**Flavonoids**	−0.1176^ns^	0.1701^ns^	−0.0947^ns^	0.2621^ns^	−0.0456^ns^
**Procyanidins**	0.9638^**^	−0.3703^ns^	0.9732^**^	0.9536^**^	0.5574^ns^
**Total tannins**	0.9923^**^	−0.3548^ns^	0.9772^**^	0.9307^**^	0.4886^ns^
**Total polyphenols**	0.9933^**^	−0.4129^ns^	0.9866^**^	0.9132^*^	0.5625^ns^

*P < 0.05; **P < 0.01; ns = not significant.
